# Novel diversity within marine Mamiellophyceae (Chlorophyta) unveiled by metabarcoding

**DOI:** 10.1038/s41598-019-41680-6

**Published:** 2019-03-26

**Authors:** Margot Tragin, Daniel Vaulot

**Affiliations:** 1 Sorbonne Université, CNRS, UMR 7144, Station Biologique, Place Georges Teissier, 29680 Roscoff, France; 2 Asian School of the Environment, Nanyang Technological University, Singapore, Singapore

## Abstract

Mamiellophyceae (unicellular green algae) are a key phytoplankton group in coastal waters. Although extensively studied over the last 20 years, the overall oceanic distribution of the major species/clades is still poorly known. To address this problem, we analyzed the 2014 Ocean Sampling Day (OSD) metabarcoding dataset providing sequences from the V4 hypervariable region of the 18S rRNA gene for 157 samples collected at 143 mostly coastal stations. Mamiellophyceae were found at nearly all OSD stations and represented 55% of the green microalgae (Chlorophyta) reads. We performed phylogenetic analyses of unique OSD metabarcodes (amplicon single variants, ASVs) and GenBank reference sequences from cultures and from the environment, focusing on the four most represented genera: *Ostreococcus* (45% of the Mamiellophyceae reads), *Micromonas* (34%), *Bathycoccus* (10%) and *Mantoniella* (8.7%). These analyses uncovered novel diversity within each genus except *Bathycoccus*. In *Ostreococcus*, a new clade (E) was the second most represented clade after *Ostreococcus* “*lucimarinus*”. *Micromonas* could be separated into nine clades, exceeding the six species and candidate species already described. Finally, we found two new environmental clades within *Mantoniella*. Each Mamiellophyceae clade had a specific distribution in the OSD dataset suggesting that they are adapted to different ecological niches.

## Introduction

In marine waters, the accepted paradigm is that the so-called “red” lineage (mainly diatoms and dinoflagellates) is dominant, while the “green” lineage (land plants) is dominant in terrestrial environments^1^. These two lineages are differentiated by the evolutionary origin of their chloroplasts: those of the “green” lineage are surrounded in most cases by two membranes, which is evidence of a single endosymbiotic event, and their major photosynthetic pigments are chlorophyll *a* and *b*^1^. Studies in coastal waters performed with both microscopic and molecular techniques found that the “green” lineage, which is mainly represented by Chlorophyta among unicellular protists, can be abundant in these ecosystems, especially among the smaller size fractions^2–4^. Metabarcoding studies following the development of high-throughput sequencing (HTS) have confirmed the importance of Chlorophyta in marine waters^5,6^.

Mamiellophyceae consists of three orders: Mamiellales, Dolichomastigales and Monomastigales^7^. Monomastigales are confined to freshwater environments, while Dolichomastigales, although quite diversified in marine waters^8,9^, are a minor component of Mamiellophyceae in coastal waters. In contrast, Mamiellales, composed of two families, Mamiellaceae and Bathycoccaceae, host some of the most common Chlorophyta microalgae such as the widely distributed *Micromonas*, the smallest known eukaryote *Ostreococcus* and the coccoid *Bathycoccus*^7^. Within Mamiellaceae, *Micromonas pusilla*^10^ was recently split into four species, namely, *Micromonas bravo* (previously clade B.E.3), *Micromonas commoda* (previous clade A.ABC.1–2), *Micromonas polaris* (previously clade B arctic), *Micromonas pusilla* (previously clade C.D.5), and two clades described as candidate species 1 (clade B._.4) and candidate species 2 (clade B warm)^11^. Within the genus *Mantoniella*, only four species have been described: the widely distributed *Mantoniella squamata*^3,12^, first described as *Micromonas squamata*^13^, *Mantoniella antarctica*^14^, and two newly described species *Mantoniella baffinensis* and *Mantoniella beaufortii* that appear restricted to arctic waters^15^. Within Bathycoccaceae, four *Ostreococcus* clades have been delineated^16^: *Ostreococcus tauri*^17^ and *Ostreococcus mediterraneus*^18^, both of which were formerly described, and *Ostreococcus* “*lucimarinus*” (clade A) and clade B, both of which lack formal taxonomic description. Analyses of pigment content and response to light levels allowed two broad ecotypes to be distinguished: strains adapted to high light (*O*. *tauri*,*O*. *mediterraneus* and *O*. “*lucimarinus*”) and those adapted to low light (*Ostreococcus* clade B)^19^. The second genus within Bathycoccaceae hosts a single species, *Bathycoccus prasinos*^20^. No clades can be delineated within this species based on 18S rRNA gene sequences from cultures and the environment. However, divergence in ITS sequences suggests that *B. prasinos* probably consists of two different species^21,22^.

The European Ocean Sampling Day (OSD) project sampled global coastal waters in 2014 at approximately the summer solstice (21 June) with the aim of analyzing the diversity and distribution of marine microorganisms^23^ especially in coastal waters using 18S rRNA metabarcodes (V4 and V9 hypervariable regions)^24^. This dataset allowed the distribution of fourteen classes of Chlorophyta to be determined^25^. Mamiellophyceae^7^, the most prevalent class in all coastal environments^26^, did not show any geographic distribution patterns or environmental preference^25^.

The present paper uses the OSD V4 datasets to analyze the taxonomic diversity and global distribution of four major Mamiellophyceae genera: *Ostreococcus*, *Micromonas*, *Bathycoccus* and the less studied *Mantoniella*. Our analyses reveal the existence of novel clades within *Ostreococcus*, *Micromonas* and *Mantoniella* and that most species/clades have specific oceanic distributions.

## Material and Methods

The OSD consortium provided two metabarcoding datasets for 2014 using the V4 region of the 18S rRNA gene: the LGC dataset (sequenced by LGC Ltd.) consisting of 157 water samples from 143 stations (see http://oceansamplingday.blogspot.com/2013/04/osd-sites_5.html) filtered through 0.22 *μ*m pore size Sterivex filters without prefiltration and the Life Watch (LW) dataset consisting of a subset of 29 water samples filtered through 0.8 *μ*m pore size polycarbonate membranes without prefiltration. The extraction, PCR and sequencing protocols were described previously^24,25^. In brief, the LGC and LW data originated from the same water samples but were processed independently for filtration, DNA extraction, PCR amplification and sequencing (both Illumina 2 × 250 bp). The LGC and LW datasets contained approximatively 5 and 9 million V4 sequences, respectively, resulting in higher coverage for the LW dataset.

The LGC and LW datasets (https://github.com/MicroB3-IS/osd-analysis/wiki/Guide-to-OSD-2014-data) were analyzed with the same pipeline using mothur software v. 1.35.1^27^. Reads were filtered to keep only sequences without ambiguities (N) and longer than 300 bp. Reads were aligned with SILVA seed release 123 reference alignment^28^ and corrected by hand to remove gaps at the beginning and at the end of alignments. The aligned datasets were filtered by removing columns containing only insertions. Chimeras were detected using Uchime v. 4.2.40^29^ as implemented in mothur. Representatives of sets of identical sequences, referred to here as Amplicon Single Variants (ASVs, following Callahan *et al*.^30^) were assigned using the Wang classifier as implemented in mothur and the PR^2^ reference database^31^ version 4.2 (https://figshare.com/articles/PR2_rRNA_gene_database/3803709/2) for which the Chlorophyta sequences had been recently curated^6^.

To confirm the assignation and explore the genetic diversity of Mamiellophyceae, ASVs from *Bathycoccus*, *Micromonas*, *Ostreococcus* and *Mantoniella* represented by more than 200 reads in either the LW or LGC dataset were selected. These selected ASVs were aligned to GenBank reference sequences using MAFFT v. 7.017^32^. Maximum likelihood (ML) phylogenies were built using FastTree v. 1.0^33,34^ as implemented in Geneious v. 7.1.9^35^. Bayesian phylogenies were built with MrBayes v. 3.2.6^36^. implemented in Geneious. Clades were defined by both the presence of clear signatures in the alignments and phylogenetic features^6,37,38^: a clade had to be monophyletic, found with the two different construction methods (i.e., ML and Bayesian) and supported by bootstrap values greater than 70 %.

We selected a subset of 92 samples with more than 100 Mamiellophyceae reads (Table S1) to compute the relative abundance of selected ASVs using R software version 3.3.1 (http://www.R-project.org/). Graphics were created using the R packages ggplot2, ComplexHeatmap^39^ and treemapify.

## Results and Discussion

We analyzed unique sequences (ASVs^30^) of two separate OSD datasets: LGC and LW. We focused on the LGC dataset which encompasses a much larger number of samples than the LW dataset which corresponds to a subset of the OSD samples that were processed in a completely independent manner compared to the LGC dataset (differences in filtration, DNA extraction, PCR and Illumina sequencing). The LW dataset was mainly used to confirm that the LGC ASVs were not artifacts by verifying that any major LGC ASV corresponded to an LW ASV with a strictly identical sequence.

**Figure 1:**
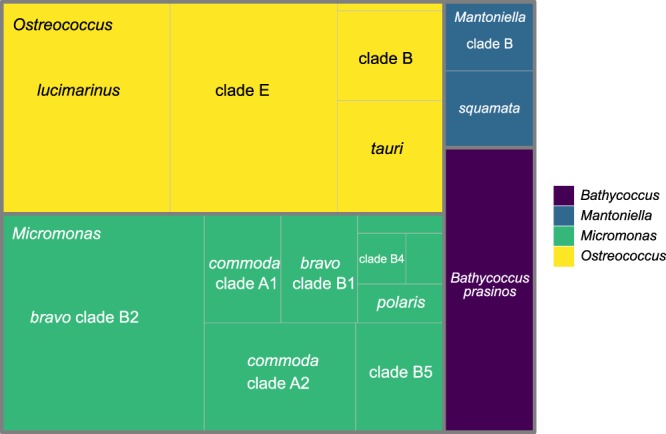
Treemap of the number of reads for Mamiellophyceae genera and clades for the OSD2014 LGC dataset. Only selected ASVs with more than 200 total reads (Table 1) and stations with more than 100 Mamiellophyceae reads (Table S1) were taken into account.

Mamiellophyceae represented 55% of the Chlorophyta reads found in the OSD 2014 surface samples^25^. Overall 10,447 Mamiellophyceae ASVs (97.5% of the ASVs) were assigned to seven genera, while the other ASVs were classified only at the family or order level. Four genera were clearly dominant: *Ostreococcus*, *Micromonas*, *Bathycoccus* and *Mantoniella*, the former two having almost equal contributions (Fig. 1). We decided to focus on 92 samples having at least 100 Mamiellophyceae reads and on 23 major LGC ASVs for the four main genera that were represented by at least 200 reads (Table 1). These major ASVs corresponded to 68% of the Mamiellophyceae reads. Among these ASVs, the one assigned to *B. prasinos* was the most widely distributed followed by two assigned to *Micromonas* clades and one assigned to *O*. “*lucimarinus*” (Fig. S1).

**Table 1:** Major Mamiellophyceae amplicon single variants (ASVs) from the LGC and LW datasets: taxonomic assignation, total abundance and representative sequences name.

ASV code	Species	Reads	Representative Sequence
ASV_LGC_00001	*Micromonas bravo* clade B2	28810	HWI-M02024_112_000000000-ACJ3F_1_2114_15858_18370
ASV_LGC_00002	*Ostreococcus lucimarinus*	22885	HWI-M02024_112_000000000-ACJ3F_1_1101_12841_20852
ASV_LGC_00003	*Ostreococcus* clade E	22720	HWI-M02024_112_000000000-ACJ3F_1_2113_19386_13300
ASV_LGC_00004	*Bathycoccus prasinos*	16750	HWIM02024_112_000000000ACJ3F_1_1110_9700_15401
ASV_LGC_00005	*Micromonas commoda* clade A2	10787	HWI-M02024_112_000000000-ACJ3F_1_1105_17215_10467
ASV_LGC_00006	*Ostreococcus* clade B	6207	HWI-M02024_112_000000000-ACJ3F_1_2104_26271_12640
ASV_LGC_00007	*Micromonas commoda* clade A1	5465	HWI-M02024_112_000000000-ACJ3F_1_2109_7000_7275
ASV_LGC_00008	*Micromonas bravo* clade B1	5459	HWI-M02024_112_000000000-ACJ3F_1_1101_14140_2642
ASV_LGC_00009	*Micromonas* clade B5	5251	HWI-M02024_112_000000000-ACJ3F_1_1106_22514_11146
ASV_LGC_00010	*Mantoniella squamata*	4500	HWI-M02024_112_000000000-ACJ3F_1_1103_8489_15072
ASV_LGC_00011	*Mantoniella* clade B	4070	HWI-M02024_112_000000000-ACJ3F_1_1101_16376_19283
ASV_LGC_00012	*Ostreococcus tauri*	3558	HWI-M02024_112_000000000-ACJ3F_1_1105_25414_3189
ASV_LGC_00013	*Ostreococcus tauri*	3288	HWI-M02024_112_000000000-ACJ3F_1_1116_6656_8393
ASV_LGC_00014	*Micromonas polaris*	2200	HWI-M02024_112_000000000-ACJ3F_1_1104_18898_13160
ASV_LGC_00015	*Micromonas* clade B4	1616	HWI-M02024_112_000000000-ACJ3F_1_1115_22402_20688
ASV_LGC_00016	*Micromonas pusilla*	1259	HWI-M02024_112_000000000-ACJ3F_1_1102_17618_11009
ASV_LGC_00017	*Micromonas* clade B3	1096	HWI-M02024_112_000000000-ACJ3F_1_1106_10729_9219
ASV_LGC_00018	*Ostreococcus tauri*	1061	HWI-M02024_112_000000000-ACJ3F_1_1101_9670_10972
ASV_LGC_00019	*Micromonas* clade B5	1045	HWI-M02024_112_000000000-ACJ3F_1_1103_25501_7171
ASV_LGC_00022	*Ostreococcus mediterraneus*	369	HWI-M02024_112_000000000-ACJ3F_1_1106_29619_14577
ASV_LGC_00023	*Ostreococcus* clade E	277	HWI-M02024_112_000000000-ACJ3F_1_1116_18277_10405
ASV_LGC_00024	*Ostreococcus mediterraneus*	248	HWI-M02024_112_000000000-ACJ3F_1_1103_20917_24371
ASV_LGC_00025	*Ostreococcus lucimarinus*	234	HWI-M02024_112_000000000-ACJ3F_1_1102_16960_7276
ASV_LW_00001	*Ostreococcus mediterraneus*	67761	M00390_102_000000000-ACEAN_1_1106_11177_14040
ASV_LW_00002	*Ostreococcus* clade E	37318	M00390_102_000000000-ACEAN_1_1107_11816_19819
ASV_LW_00003	*Micromonas polaris*	35194	M00390_102_000000000-ACEAN_1_2105_19731_21730
ASV_LW_00004	*Mantoniella squamata*	25692	M00390_102_000000000-ACEAN_1_1107_20899_6104
ASV_LW_00005	*Bathycoccus prasinos*	20779	M00390_102_000000000-ACEAN_1_1101_8267_5862
ASV_LW_00006	*Micromonas bravo* clade B2	20153	M00390_102_000000000-ACEAN_1_1101_13226_2947
ASV_LW_00007	*Micromonas commoda* clade A2	14869	M00390_102_000000000-ACEAN_1_1101_19723_8016
ASV_LW_00008	*Ostreococcus tauri*	13511	M00390_102_000000000-ACEAN_1_1101_10242_22531
ASV_LW_00009	*Ostreococcus lucimarinus*	7310	M00390_102_000000000-ACEAN_1_1101_22616_1270
ASV_LW_00010	*Ostreococcus tauri*	5640	M00390_102_000000000-ACEAN_1_1101_13071_3223
ASV_LW_00011	*Micromonas pusilla*	2806	M00390_102_000000000-ACEAN_1_1101_17876_4103
ASV_LW_00012	*Micromonas commoda* clade A1	2610	M00390_102_000000000-ACEAN_1_1101_13887_6615
ASV_LW_00013	*Micromonas bravo* clade B1	2357	M00390_102_000000000-ACEAN_1_1108_21831_3480
ASV_LW_00014	*Micromonas* clade B3	1862	M00390_102_000000000-ACEAN_1_1112_28497_17492
ASV_LW_00015	*Micromonas* clade B4	1078	M00390_102_000000000-ACEAN_1_1101_13697_13215
ASV_LW_00016	*Mantoniella* clade B	935	M00390_102_000000000-ACEAN_1_1101_10638_9605
ASV_LW_00017	*Ostreococcus tauri*	908	M00390_102_000000000-ACEAN_1_1101_19812_1055
ASV_LW_00018	*Mantoniella* clade A	841	M00390_102_000000000-ACEAN_1_1115_21286_22319
ASV_LW_00020	*Micromonas polaris*	668	M00390_102_000000000-ACEAN_1_1101_14320_1948
ASV_LW_00021	*Micromonas* clade B5	661	M00390_102_000000000-ACEAN_1_1101_13671_3127
ASV_LW_00022	*Micromonas commoda* clade A2	462	M00390_102_000000000-ACEAN_1_1104_4249_10680
ASV_LW_00024	*Ostreococcus* clade B	366	M00390_102_000000000-ACEAN_1_1113_12543_2229
ASV_LW_00027	*Micromonas bravo* clade B1	205	M00390_102_000000000-ACEAN_1_1103_16789_17810

### *Ostreococcus*

**Figure 2:**
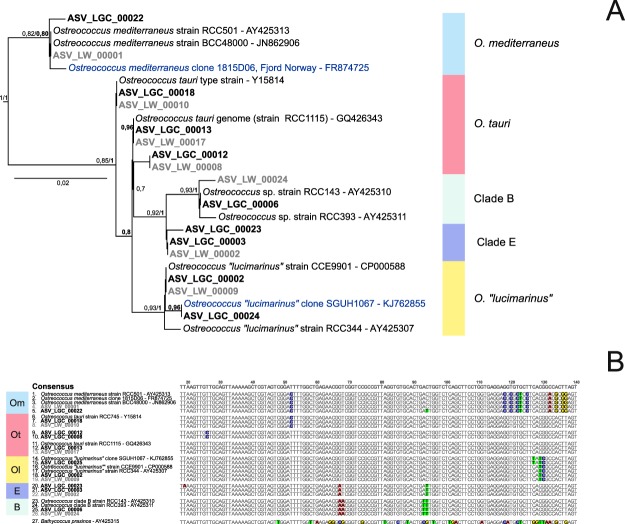
Phylogenetic diversity within the genus *Ostreococcus*. (**A**) Phylogenetic tree of 26 *Ostreococcus* sequences from the V4 region of the 18S rRNA gene (FastTree). The tree was rooted with *B*. *prasinos*. Only ML bootstrap values higher than 70% are represented. Bayesian posterior probabilities are in bold. ASVs are in bold: black for the LGC dataset and gray for the LW dataset. Only ASVs represented by more than 200 reads were taken into account. Environmental GenBank sequences are in blue. (**B**) Alignment of 26 *Ostreococcus* V4 regions: the alignment is 341 bp long, but only the main signatures are shown (between positions 20 and 140 of the original alignment).

**Figure 3:**
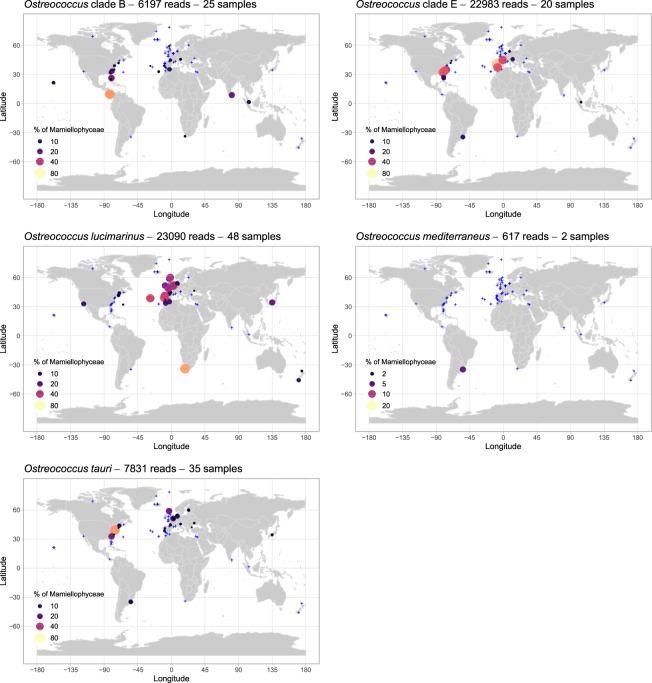
Distribution of the major *Ostreococcus* ASVs for OSD2014 (LGC). The three major *O*. *tauri* ASVs have been pooled together. Circle surface corresponds to the percentage of ASV reads relative to the total number of Mamiellophyceae reads. Samples for which the contribution of the ASV was lower than 1% or where it was absent are represented by blue crosses. A zoomed-in map of European waters is provided in Fig. S3.

In total, 4,223 ASVs from the LGC dataset were assigned to the genus *Ostreococcus*, among which 10 had more than 200 reads (Table 1). For each of these abundant LGC ASVs, we found a corresponding ASV in the LW dataset. These sequences constituted five clades (Fig. 2A), four of which were previously described and a new one that we named clade E, following the previous conventions for naming *Ostreococcus* clades^16^. The same tree topology was recovered with ML and Bayesian methods. Alignments (Fig. 2B) confirmed that the V4 region exhibited clear signatures delineating the five *Ostreococcus* clades. In this region, the genetic variation between clades (Table S2) was too low to discriminate the different clades when V4 operational taxonomic units (OTUs) are built with a 99% identity threshold, except for *O. mediterraneus* (98.3%). In terms of overall distribution, *Ostreococcus* was completely absent from high latitudes beyond 65° N (Iceland, Greenland and Nunavut, Fig. 3). *O*. “*lucimarinus*” was the most widely distributed (Fig. S1). In general none of the different *Ostreococcus* species/clades seemed to co-occur, with the exception of *O. tauri* and *O*. “*lucimarinus*” and to a lesser extent, clades B and E (Fig. S2).

Three ASVs were assigned to the first described species of *Ostreococcus*, *O. tauri*, with a number of reads ranging from 1,000 to 3,500 (Table 1). These ASVs did not form a monophyletic clade in either ML and Bayesian tree (Fig. 2A). The two major ASVs (ASV_LGC_00012 and ASV_LGC_00013) were closely related to the 18S rRNA sequence recovered from the genome of *O. tauri* (GQ426344). The third ASV (ASV_LGC_00018) was identical to the sequence with accession number Y15814 which was obtained more than 20 years ago for the type strain of *O. tauri* isolated from the Thau Lagoon on the French Mediterranean coast^17^. The V4 alignment (Fig. 2B) revealed two signatures for *O. tauri*. *Ostreococcus tauri* ASVs represented more than 1% of Mamiellophyceae reads at 35 stations and were abundant on the East Coast of the USA, and in the Baltic Sea, the Adriatic Sea (Venice Lagoon), the Black Sea and Uruguay lagoons (Figs 3 and S3). Some of these samples corresponded to brackish environments (e.g., a salinity 9.0, 7.4 and 7.2 at OSD35, OSD36 and OSD186, respectively, all in Chesapeake Bay, USA or of 24 at OSD39 in Charleston Harbor, USA) which were similar to those from Thau Lagoon where salinity is highly variable from 24 to 38^17^. Surprisingly *O. tauri* was absent from the western Mediterranean Sea despite the fact that strains have been isolated from there^18^. The existence of several ASVs suggests that *O. tauri* might be a complex of species, that need to be better distinguished and that could be adapted to different ranges of salinity.

Two major ASVs (ASV_LGC_00002 and ASV_LGC_00025) were assigned to *O*. “*lucimarinus*”, which was initially described as a high-light-adapted clade^19^, corresponding to 23,119 reads (Table 1) and representing up to 64% of the Mamiellophyceae reads off South Africa (Robben Island, OSD133). This clade dominated Atlantic and North Sea European coastal stations (Belgium, OSD183: 62% and OSD184: 44%; Portugal OSD115: 53%) and represented 40% of Mamiellophyceae reads at one of the three Azores stations (OSD98, Fig. 3). In contrast, *O*. “*lucimarinus*” was almost absent from the Mediterranean Sea and tropical waters (Figs 3 and S3). This distribution is consistent with the clade being a cold mesotrophic coastal clad, based on observations by qPCR (clade OI according to Demir-Hilton *et al*.^40^, see below).

A single ASV was assigned to *Ostreococcus* clade B, initially described as a low-light-adapted clade^19^, representing 6,207 reads, and accounting for 62% of the Mamiellophyceae reads off Panama (OSD51, Fig. 3). Clade B constituted more than 10% of the Mamiellophyceae reads at seven tropical and subtropical stations from various oceans (OSD60 in South Carolina; OSD25, OSD37 and OSD51 in Florida; OSD95 in Singapore; OSD122 in the Red Sea; OSD147 in Sri Lanka, Fig. 3), which is consistent with previous results obtained by qPCR which detected clade B at warm oligotrophic sites^40,41^.

The novel *Ostreococcus* clade E was represented by a single ASV in the LGC dataset (22,720 reads) and an identical sequence was also found in the LW dataset, suggesting that the detection of this clade was not an artefact. The clade E sequence is very similar to that of clade B, with two clear bp differences (Fig. 2B), and is 100% similar to a single sequence available from GenBank (accession number MH008654) also obtained by Illumina sequencing from South China Sea waters^42^. The V4 sequence of clade E is 99.4% similar to that of clade B, such that these two clades may have been lumped together in many metabarcoding studies that considered OTUs rather than ASVs. Clade E could be locally dominant in OSD samples representing up to 70% of the Mamiellophyceae reads (OSD111, off Portugal). The clade dominated coastal warm temperate stations (Fig. 3) on both sides of the Atlantic Ocean (Southern USA: OSD39, OSD58 and OSD143; Portugal: OSD81, OSD111, OSD117 and OSD153; and France: OSD154) and the Mediterranean Sea (Adriatic Sea off Venice: OSD69). It is surprising that no culture has been obtained for this new clade, but this absence could be due to the fact that the clade requires specific conditions to grow.

Finally, two ASVs with a low number of reads (369 and 248, in the LGC dataset, Table 1) were assigned to the species *O. mediterraneus*. These ASVs were found only in a lagoon along the coast of Uruguay (5% and 6% of Mamiellophyceae reads at OSD149 and OSD150, respectively, Fig. 3). This distribution is consistent with the fact that almost all strains of *O. mediterraneus* have been isolated from coastal lagoons along the Mediterranean Sea coast^18^, suggesting that this species is restricted to very specific environments with fluctuating salinity. Interestingly, a sequence matching *O. mediterraneus* has also been found in the freshwater Lake Taihu (China)^43^.

Two sets of qPCR primers and probes were previously designed^40^ based on available V4 sequences from strains in culture in order to discriminate two *Ostreococcus* groups (OI and OII). The OI set targets *O*. “*lucimarinus*” but also recognizes *O. tauri*^40^, which has two mismatches with the reverse primer (Fig. S4), while the OII group targets *Ostreococcus* clade B. Interestingly the new *Ostreococcus* clade E has four mismatches with set OI (one with the forward primer, one with the probe and two with the reverse primer) and two mismatches with set OII (one with the forward primer and one with the probe). Since some of these mismatches are located on the 3′ end of the forward primer (Fig. S4), these sets may be unable to recognize *Ostreococcus* clade E. If strains from *Ostreococcus* clade E become available, it would probably be necessary to design new qPCR sets specific to each of the *Ostreococcus* species/clades, which seem to have different distributions (Fig. 3).

### *Micromonas*

**Figure 4:**
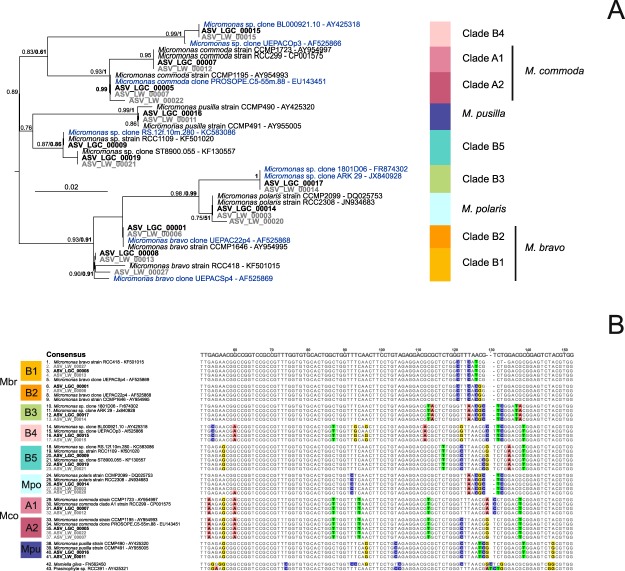
Phylogenetic diversity within the genus *Micromonas*. (**A**) Phylogenetic tree of 39 *Micromonas* sequences from the V4 region of the 18S rRNA gene (FastTree). The tree was rooted with Mamiellales (RCC391, AY425321 and *Mamiella gilva*, FN562450). Legend as in Fig. 2. (**B**) Alignment of 39 *Micromonas* V4 regions: the alignment is 327 bp long, but only the main signatures are shown (between positions 50 and 150 of the original alignment).

A total of 4,285 unique OSD LGC sequences were assigned to the genus *Micromonas*, with 10 corresponding to more than 200 reads. Phylogenetic analysis and 18S V4 signatures allowed these sequences to be divided into 9 major clades (Fig. 4) corresponding to the species, candidate species and clades recently described by Simon *et al*.^11^, with the exception of a new subarctic clade not seen previously. *M. commoda* and *M. bravo* were each divided further into two subclades (Table 2). The clade nomenclature of *Micromonas* has been very confusing, with at least 4 different conventions used (Table 2). We decided to adopt a novel clade nomenclature that relies on the original three clades of Guillou *et al*.^16^ (A, B, and C), appending a number for subclades (e.g., B3, B4, B5). This scheme has been used for various taxonomic groups and allows new clades and subclades to be added easily^26^.

**Table 2:** Equivalence between the clade nomenclature for the genus *Micromonas* in this paper compared to those of Guillou *et al*.^16^, Slapeta *et al*.^48^, Worden^72^ and Simon *et al*.^11^.

Genus	Species	This paper	Guillou 2004	Slapeta 2006	Worden 2006	Simon 2017
*Micromonas*	*commoda*	A1	A	A	A.A.2	*commoda*
	*commoda*	A2	A	BC	A.BC.1	*commoda*
	*bravo*	B1	B	E	B.E.3	*bravo*
	*bravo*	B2	B	E	B.E.3	*bravo*
	*sp*.	B3	B			
	*sp*.	B4	B		B._.4	candidate species 1
	*sp*.	B5	B			candidate species 2
	*polaris*		B	E	B.E.3	*polaris*
	*pusilla*		C	D	C.D.5	*pusilla*

A well-supported tree topology was recovered with both ML and Bayesian methods. The genetic divergence between clades was greater than 1% for almost all the clade pairs (Table S3), allowing all clades to be distinguished with the 99% identity threshold commonly used in metabarcoding studies, except for *M. commoda* A1 and A2 (99.4% identity), *M. bravo* B1 and B2 (99.3% identity), *M. polaris* and the new clade B3 (99.2%). *M. bravo* B2 was present in the largest number of samples (76, Fig. S1A). Most species/clades did not co-occur, with the exception of *M. bravo* B1 and B2 (Fig. S2).

**Figure 5:**
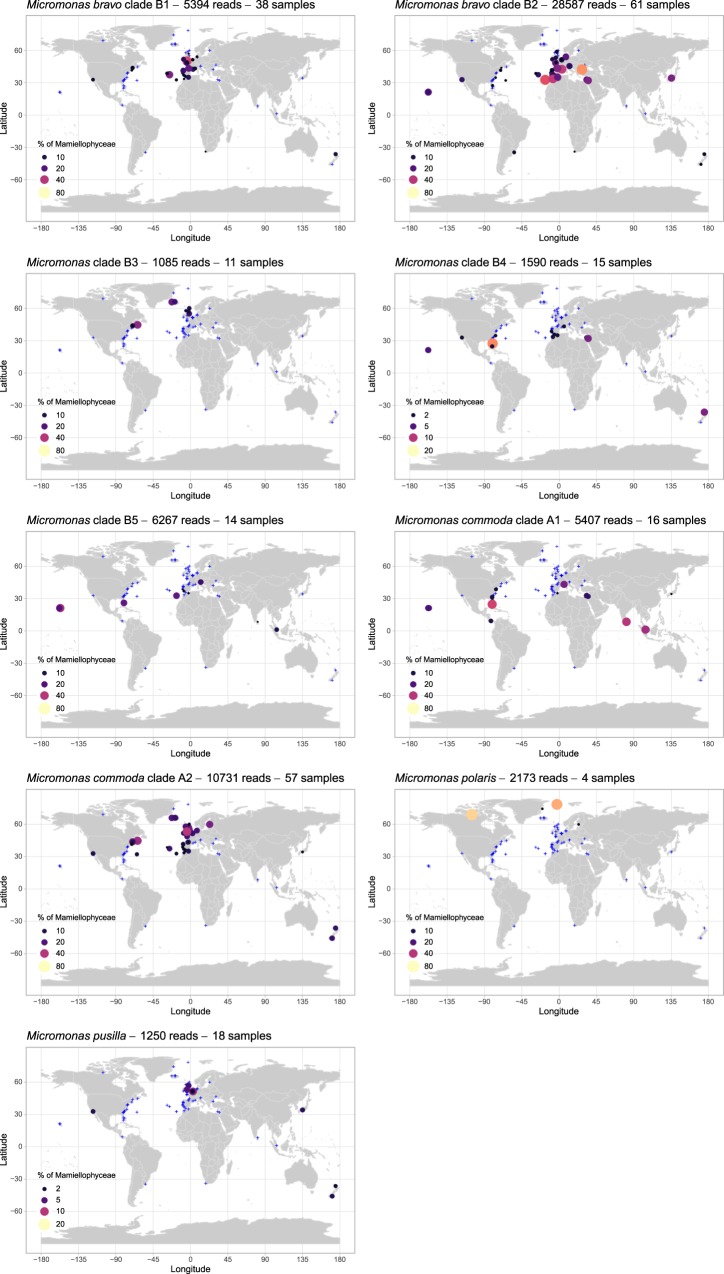
Distribution of the major *Micromonas* ASVs for OSD2014 (LGC). Legend as in Fig. 3. A zoomed-in map of European waters is provided in Fig. S5.

The major *Micromonas* ASV, represented by 28,810 reads, was assigned to *M. bravo* B2 (Fig. 4). *M. bravo* is a newly described species^11^ that was previously part of the B clade^16^. This ASV represented up to 60% of the Mamiellophyceae reads (Figs 5 and S5) in the Black Sea (OSD131) and off Portugal (OSD102). This ASV dominated most Mediterranean Sea stations, some North European stations and, to a lesser extent, some Pacific stations (14% off California at OSD43 and 28% at Hawaii OSD144). This ASV was the only *Micromonas* ASV that represented more than 10% of Mamiellophyceae reads off Japan (29% for OSD124). *M. bravo* B1 ASV (5,459 reads) represented more than 10% of Mamiellophyceae reads at three stations along the European coast (in particular 47% in the English Channel off Plymouth, OSD1).

Two LGC ASVs with a single base pair difference (Fig. 4B) were closely related to the reference sequence of “candidate species 2” from Simon *et al*.^11^ and of “clade VI” from Lin *et al*.^44^. These ASVs are referred to here as *Micromonas* sp. clade B5. These two ASVs (6,296 reads) accounted for more than 10% of the Mamiellophyceae reads at eight stations in tropical or warm waters (Fig. 5): at the three Hawaii stations (OSD56, OSD57, and OSD144), off Florida (OSD37), off Portugal (OSD101), off Singapore (OSD95) and in Venice Lagoon (OSD69 and OSD70). This distribution was consistent with data from available GenBank sequences^11^: one representative strain was isolated in the Mediterranean Sea in summer (RCC1109) and environmental clones weren recovered in the Red Sea^45^, in the South China Sea (“unknown clade”^46^) and off Taiwan (*Micromonas* clade VI^44^).

*M. commoda* A2 ASV (10,787 reads) represented more than 1% of the Mamiellophyceae reads at 57 stations (Fig. S1) and up to 35% off the Atlantic coast of Canada (OSD152). This ASV was found in the North Atlantic up to Iceland and off New Zealand (Figs 5 and S5). In contrast, this ASV was almost completely absent from the Mediterranean Sea as well as from tropical stations. The frequency of *M. commoda* A1 (5,465 reads) was above 1% at a much lower number of stations (16, Fig. 1) and accounted for up to 40% of the Mamiellophyceae reads off Sri Lanka (OSD147). *M. commoda* A1 was distributed in tropical and subtropical waters (Figs 5 and S5), in particular, off Florida, Singapore and Hawaii as well as in the Eastern Mediterranean Sea off Israel. *M. commoda* was described by Van Baren *et al*.^47^ who mentioned only that this species had not been recorded yet at high latitudes (beyond 60 North or 60 South). The species was then revised by Simon *et al*.^11^, who described the distribution of this species as ubiquitous based on available GenBank sequences. The genetic variability within this species had already been highlighted previously^48,49^ and was confirmed by the OSD data since the two subclades A1 and A2 had clearly distinct distributions. Simon *et al*.^11^ proposed the hypothesis that speciation events may be ongoing within *M. commoda*.

The ASV (1,616 reads) corresponding to *Micromonas* sp. clade B4 (“candidate species 1” according to Simon *et al*.^11^) reached a frequency of 15% off Florida (OSD29) and was found in rather warm waters (off Hawaii, Israel, and Morocco, Fig. 5), which matches the distribution of sequences available in GenBank that had been previously recovered from the Mediterranean and Red Seas as well from the Pacific Ocean^11^.

*M. polaris* ASV (2,200 reads) was the major Mamiellophyceae contributor (Fig. 5) at two stations in Arctic waters (73% in Nunavut OSD105, 66% in Fram Strait OSD146) and was also present in the Gulf of Finland (OSD30). This pattern is consistent with current knowledge of this species. *M. polaris* was first isolated from the Arctic Ocean^50^ and shown to be the dominant picoeukaryote in the Beaufort Sea in the summer^51^. This ASV was also recently recorded in the Southern Ocean^52^, although its presence appears to be less prevalent since it is absent from environmental Antarctic clone libraries^6^. A new *Micromonas* clade closely related to *M. polaris* (B3, 1,096 reads) had a maximum contribution off Canada (32% in Bedford Basin OSD152) and represented more than 10% of Mamiellophyceae reads at four subarctic stations off Maine and Iceland as well as at a temperate location off the UK coast in the North Sea (Fig. 5). This ASV is 100% similar to GenBank sequences recently obtained in the White Sea^53^.

Finally, the ASV corresponding to the first described *Micromonas* species, *M. pusilla*, was found at low abundance (1,259 reads) mostly in temperate locations (Fig. 5) corresponding to the environment from which it was initially described and isolated (e.g. CCMP490 isolated from Woods Hole, USA, and CCMP491 from the English Channel, UK^48^).

### *Bathycoccus*

**Figure 6:**
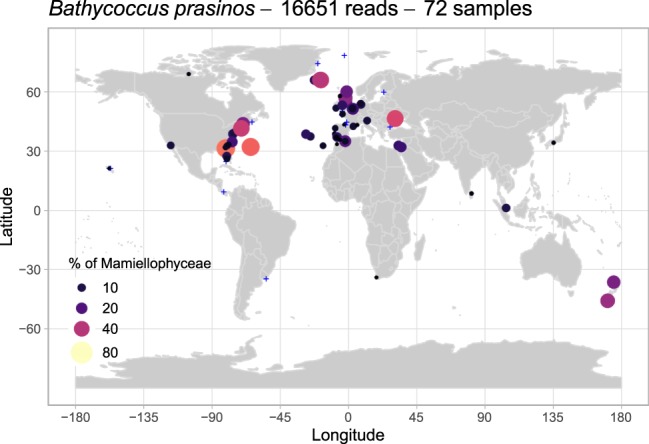
Distribution of the major *Bathycoccus* ASV for OSD2014 (LGC). Legend as in Fig. 3. A zoomed-in map of European waters is provided in Fig. S6.

The ASV corresponding to *Bathycoccus* was both the most abundant (24,391 reads) and the most prevalent, accounting for more than 1% of the Mamiellophyceae reads at 72 stations (Fig. S1) distributed all over the coastal ocean from tropical to polar waters (Figs 6 and S6). This worldwide distribution of the genus matches what has been observed based on the Tara *Oceans* dataset^21^ where the metagenome of *Bathycoccus* was recovered at a wide range of stations. *Bathycoccus* is now known to be composed of two cryptic species with identical 18S rRNA sequences (that therefore cannot be distinguished in the OSD dataset) but differences in the ITS sequences as well as at the genomic level^21,54^. The distribution of these two cryptic species (BI-genome RCC1105 and BII-genome TOSAG39-1) determined by metagenomic analysis and qPCR suggest that BI could be coastal and extending to polar waters, while BII is adapted to warmer oceanic waters^21,22,55,56^.

### *Mantoniella*

**Figure 7:**
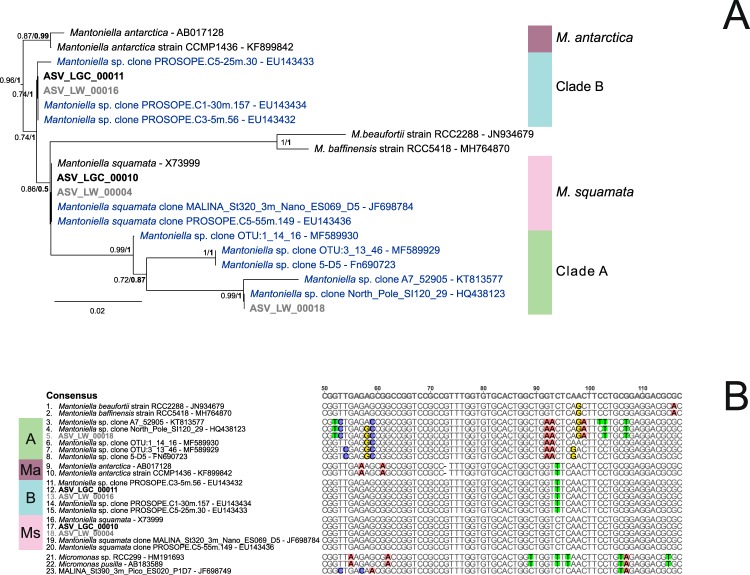
Phylogenetic diversity within the genus *Mantoniella*. (**A**) Phylogenetic tree of 19 *Mantoniella* sequences from the V4 region of the 18S rRNA gene (FastTree). The tree was rooted with the 3 *Micromonas* sequences (AB183589, HM191693, and JF698749). Legend as in Fig. 2. (**B**) Alignment of 19 *Mantoniella* V4 regions, the alignment was 368 bp long, but only the main signatures are shown (between the 20th and 141st position of the original alignment).

Two ASVs with more than 200 reads were assigned to the genus *Mantoniella*, corresponding to a total of 8,570 reads (Table 1). Except for the morphological species *M. squamata*^3,12^ and *M. antarctica*^14^, no clades based on 18S rRNA gene sequences have been yet described^7,16^. However, the V4 hypervariable region of the 18S rRNA gene examined using publicly available reference sequences as well as OSD ASVs highlights two new *Mantoniella* clades, which we named A and B, both with well-supported phylogenies (Fig. 7A) and clear sequence signatures (Fig. 7B).

**Figure 8:**
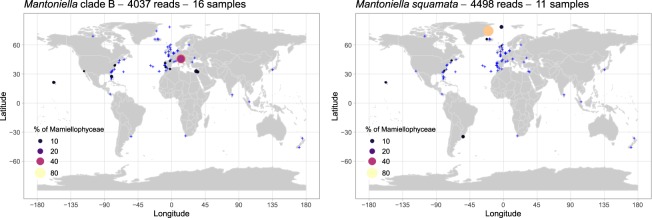
Distribution of the major *Mantoniella* ASVs for OSD2014 (LGC). Legend as in Fig. 3. A zoomed-in map of European waters is provided in Fig. S7.

The most abundant ASV corresponded to the species *M. squamata* (4,500 reads) and represented up to 70% of the Mamiellophyceae reads off Greenland (OSD80, Figs 8 and S7), while at all other stations it always accounted for less than 10%. However, this ASV was found at stations with very different environmental conditions, including off Hawaii and in a lagoon on the coast of Uruguay (Fig. 8). This finding is consistent with previous descriptions of *M. squamata* as a cosmopolitan species^3^ and several studies reporting in particular its presence in northern high latitudes^51,57^.

The ASV corresponding to *Mantoniella* clade B (4,070 reads) was more widespread, representing more than 1% of the Mamiellophyceae reads at 16 stations (Fig. S1), especially in Venice Lagoon (53% at OSD47, 36% at OSD70 and 12% at OSD69) but also in other moderately warm waters (eastern Mediterranean Sea, Gibraltar, California, Hawaii, Figs 8 and S7), matching GenBank sequences previously found in the Mediterranean Sea^8^.

No ASV corresponding to *Mantoniella* clade A was found in the LGC dataset but one ASV from the LW dataset (841 reads) matched this clade. This clade was present only off Greenland (OSD80) which matches the environment where other GenBank sequences from this clade have been obtained (sea ice from the Arctic Ocean^58^, the White Sea^53^ and the Baltic Sea^59^), suggesting that *Mantoniella* clade A is an ice alga.

Finally, no major ASV corresponding to the species *M. antarctica* was found in either the LGC or LW dataset. In particular, this species was absent from the single station sampled in Antarctica (OSD187, Palmer Station) in the middle of the austral winter, where only 85 Mamiellophyceae reads were recorded. Similarly, no major ASVs corresponding to the newly described species *M. baffinensis* and *M. beaufortii* were found in either the LGC or LW datasets, although a minor ASV corresponding to the latter species was found off Greenland (OSD80)^15^.

## Limitations of the OSD Dataset

The OSD metabarcoding dataset is invaluable to determining the distribution of many phytoplankton groups in coastal waters^25,26,60^. However, it must be always emphasized that metabarcoding is not a quantitative method because of biases in PCR and because of the variation in the number of 18S rRNA gene copies per organism^61^. In the case of Mamiellophyceae, because they are small, the number of copies is low^61^ and does not vary greatly between the different genera (e.g. two and four copies in *Bathycoccus* and *Ostreococcus*, respectively^62,63^). This low variation supports the use of relative number of reads as a semiquantitative proxy of Mamiellophyceae contributions. Another potential problem of the OSD dataset is the fact that sampling was conducted everywhere on almost the same date (in most cases on June 21). Such simultaneous sampling has the advantage of providing a snapshot of the global coastal ocean, which allows analysis of spatial distributions without the impact of seasonality, in contrast to oceanographic expeditions such as Tara *Oceans*^5^, which sampled different ecosystems at different times of the year. One limitation of OSD is that the Northern and Southern Hemispheres were sampled at opposite points of the yearly cycle: at the summer and winter solstices, respectively. However, since sampling was mostly conducted in the Northern Hemisphere, this limitation has a low impact on data interpretation. The existence of phytoplankton cycles driven by temporal changes and nutrient cycles in coastal environments^64–66^ may explain why some species were not found in the OSD dataset in regions where strains or clones corresponding to the same species have been isolated before. As an example, *Bathycoccus* initially isolated from the Gulf of Naples in the Mediterranean Sea^20^, was not recovered at the Naples OSD station (OSD4), where only 2 Mamiellophyceae reads were obtained. Metabarcoding analyses at the Long Term Ecological Research station in the Gulf of Naples showed that Mamiellophyceae were not found in June^67^, which may explain why they were absent from the OSD dataset. In contrast, analysis of several time series^68^ led to the conclusion that *M. bravo* (previously non Arctic B.E.3 clade) dominated the *Micromonas* community in summer and should be adapted to warm well-lit coastal waters which is consistent with what we observed in the OSD dataset for which sampling was performed in June.

## Conclusion

Determining the biogeographical distribution of phytoplankton species is critical for understanding their ecophysiology in order to determine how they will adapt to predicted climate change and how marine food webs will evolve. Although Mamiellophyceae are clearly the dominant group of green algae in coastal waters^25,26^, previous analysis could not relate their relative abundance to any environmental variable^25^. Even analysis at the genus level is not sufficient to detect biogeographical patterns. For example, the genus *Micromonas* is found at virtually all OSD stations (Fig. 5). The only genus that is not widely distributed is *Ostreococcus*, which is absent from polar regions. In contrast, analysis at the species/clade levels allows some very clear patterns, in particular with respect to latitudinal distribution (Table 3), to be detected. Some species/clades have quite restricted latitudinal ranges, e.g., *M. polaris* was found only in Arctic samples, *M. pusilla* was found only in temperate waters, and *Micromonas* sp. clade B5 was found only in warm waters. Others are much more widespread, for example, *Ostreococcus* clade B, which extends from temperate to tropical waters. Some taxa seem to be restricted to specific habitats, in particular, *O. mediterraneus* is restricted to coastal lagoons. The case of the Mediterranean Sea is also interesting. This sea has been previously shown to harbor specific taxonomic groups such as Chlorodendrophyceae^25^, clade A6 of Chloropicophyceae^26^ or the Bolidophyceae *Triparma mediterranea*^69^. Here, we found that some temperate species such as *M. pusilla* or *Ostreococcus* clade E were absent from this region, but we did not find any species/clade restricted to it.

**Table 3:** Summary of the coastal distribution of Mamiellophyceae species and clades.

Genus	Species	Clade	Samples	Polar	Sub-polar	Temperate	Tropical	Med Sea	Lagoon
*Bathycoccus*	*prasinos*		72		+	+	+	+	+
*Mantoniella*	*squamata*		11	+		+	+		
	*sp*.	A	1	+					
	*sp*.	B	16			+	+	+	+
*Micromonas*	*commoda*	A1	16				+	+	
	*commoda*	A2	57		+	+			
	*bravo*	B1	38			+		+	
	*bravo*	B2	61			+		+	+
	*sp*.	B3	11		+				
	*sp*.	B4	15			+	+	+	
	*sp*.	B5	14				+		+
	*polaris*		4	+					
	*pusilla*		18			+			
*Ostreococcus*	*lucimarinus*		48		+	+			
	*sp*.	B	25			+	+	+	+
	*tauri*		35			+			+
	*mediterraneus*		2						+
	*sp*.	E	20			+			+

The column “Samples” indicates the number of samples where the species/clade represented more than 1% of Mamiellophyceae reads in the LGC dataset. For *Mantoniella* clade A was only oberved in the LW dataset. Crosses correspond to the habitat where a given species/clade is present.

Genetic diversity is quite different between the four genera that we examined. While *Bathycoccus* is composed of a single clade, at least based on the 18S rRNA gene, *Micromonas* seems to be extremely diversified with a large number of clades. Although most of the clades found in OSD have been observed before, we uncovered some new diversity. One species, *M. bravo*, was split into two subclades, namely, B1 and B2, that seem to have distinct distributions. Two new clades were uncovered: *Micromonas* sp. clade B3, which seems to be restricted to a specific latitudinal band (approximately 45° to 65° N), and *Ostreococcus* clade E which is very interesting since it is very abundant and has a distinct distribution from the closely related *Ostreococcus* clade B. Specifically, *Ostreococcus* clade E is restricted to temperate waters, while clade B is found in tropical waters. Since some oceanic regions are covered by the OSD dataset, it is possible that yet undiscovered Mamiellophyceae clades/species exist, especially in the Southern Hemisphere. To obtain more information on the new clades reported here and to determine whether they correspond to potentially novel species, several strategies are possible. First, now that the geographical distribution of these taxa is being uncovered, we may target specific environments and obtain isolates. Such targeting will allow analysis of finer resolution markers such as the ITS^11^ and determination of physiological preferences, for example, in terms of temperature^70^. Another strategy for examining clades that are hard to isolate in culture would be to determine longer sequences of the ribosomal operon, including in particular the ITS region, directly from the environment either by long-amplicon PCR using novel technologies such as nanopore sequencing^71^ or by extracting them from existing metagenomics datasets such as those obtained during the Tara Oceans project^21^.

## Supplementary information

Supplementary Material

## Data Availability

Scripts for mothur and R, and Mamiellophyceae OTU sequences, alignments, assignation and abundance for the LGC and LW datasets are provided as Supplementary Files at https://github.com/vaulot/Paper-2018_Tragin_Mamiellophyceae-R-scripts (10.6084/m9.figshare.7233110). The R processing script is detailed at https://vaulot.github.io/papers/OSD_Mamiello.html.
