# Supplementary material for: Novel diversity within marine Mamiellophyceae (Chlorophyta) unveiled by metabarcoding
Source: Sci Rep. 2019 Mar 26;9:5190. doi: 10.1038/s41598-019-41680-6 (PMC6435750; doi:10.1038/s41598-019-41680-6)
Supplement: Supplementary file 1 — Supplementary Material [file 41598_2019_41680_MOESM1_ESM.pdf]

Novel diversity within marine Mamiellophyceae (Chlorophyta) unveiled by metabarcoding

Supplementary material

Margot Tragin[1]
Daniel Vaulot[1,2]

[1]Sorbonne Université, CNRS, UMR 7144
Station Biologique, Place Georges Teissier, 29680 Roscoff, France

[2]Asian School of the Environment, Nanyang Technological University, Singapore

Date: February 15, 2019

All supplementary data have been deposited to

GitHub : https://github.com/vaulot/Paper-2018_Tragin_Mamiellophyceae-R-scripts

(DOI: 10.6084/m9.figshare.7233110)

Supplementary data

Supplementary Data S1: (OSD_mamiello_mothur.sh) Mothur script for sequence analysis.

Supplementary Data S2: (OSD_Mamiello.html). R script for generating distribution maps and heatmaps with output.

Supplementary Data S3: (Excel file OSD_Mamiello_Tables Supplementary.xlsx). Description of the different sheets:

- ”metadata”: list of OSD stations with coordinates and metadata.
- ”samples”: list of OSD samples.
- ” otus LGC Mamiello”: Mamiellophyceae LGC ASV table with taxonomy and number of reads assigned to each ASV per OSD 2014 samples.
- ” otus LW Mamiello”: Mamiellophyceae LW ASV table with taxonomy and number of reads assigned to each ASV per OSD 2014 samples.
- ”sequences GenBank”: Genbank accession numbers for sequences used to build the trees.
- ”simil_Ostreococcus”: Matrix of the pairwise percent identity between *Ostreococcus* clades. The calculation was done based on the alignment available as Supplementary data.
- ”simil_Micromonas”: idem for *Micromonas*.
- ”simil_Mantoniella”: idem for *Mantoniella*.

Supplementary Data S4: (OSD_Mamiello_ASV_LGC.fasta) OSD 2014 Mamiellophyceae ASV representative sequences (LGC sequencing).

Supplementary Data S5: (OSD_Mamiello_ASV_LW.fasta) OSD 2014 Mamiellophyceae ASV representative sequences (LW sequencing).

Supplementary Data S6: (OSD_Mamiello_ASV_LGC_Ostreococcus_align.fasta) Alignment of major LGC and LW *Ostreococcus* ASV with related GenBank sequences from clones and cultures.

Supplementary Data S7: (OSD_Mamiello_ASV_LGC_Micromonas_align.fasta) Idem for *Micromonas*.

Supplementary Data S8: (OSD_Mamiello_ASV_LGC_Mantoniella_align.fasta) Idem for *Mantoniella*.

List of supplementary tables

Table S1 List of the 92 OSD samples used in this paper. Only samples with more than 100 Mamiellophyceae LGC reads were taken into account. The percentage of Mamiellophyceae is relative to the total number of LGC reads at each station.

Table S2 Matrix of the pairwise percent identity between *Ostreococcus* clades. The calculation was done based on the alignment available as Supplementary data S6.

Table S3 Matrix of the pairwise percent identity between *Micromonas* clades. The calculation was done based on the alignment available as Supplementary data S7.

Table S4 Matrix of the pairwise percent identity between *Mantoniella* clades. The calculation was done based on the alignment available as Supplementary data S8.

List of supplementary figures

Fig. S1 A. Percentage of OSD samples where a given ASV was detected (at least one read). Numbers at right of bars correspond to number of samples. B. Idem but for samples where the ASV contributed more than 1% of the Mamiellophyceae reads. Stations with less than 100 Mamiellophyceae reads were not considered. The three major *O. tauri* ASVs have been pooled together.

Fig. S2 Heatmap of the Mamiellophyceae communities in OSD samples. The number of read corresponding to the major ASVs were normalized by the number of Mamiellophyceae reads per station. Stations with less than 100 Mamiellophyceae reads were not considered.

Fig. S3 Distribution of major *Ostreococcus* ASVs for OSD2014 (LGC) in European waters. The three major O. tauri ASVs have been pooled together. Circle surface corresponds to the percentage of ASV reads relative to the total number of Mamiellophyceae reads. Samples for which the ASV contribution was lower than 1 % are represented by blue crosses.

Fig. S4 Alignments of *Ostreococcus* V4 regions and localization of the qPCR primers and probes (Demir-Hilton et al. 2011) used to quantify the OI (A) and OII (B) clades. Sequences with no mismatches to each probe set are in bold. Blue and green sequences correspond to LGC and LW datasets, respectively.

Fig. S5 Same as Fig. S3 for *Micromomas*.

Fig. S6 Same as Fig. S3 for *Bathycoccus*.

Fig. S7 Same as Fig. S3 for *Mantoniella*.

Table S1. List of the 92 OSD samples used in this paper. Only samples with more than 100 Mamiellophyceae LGC reads were taken into account. The percentage of Mamiellophyceae is relative to the total number of LGC reads at each station.

Station	Depth (m)	Name	Country	Ocean	Mamiellophyceae reads	Mamiellophyceae %
OSD1	0	Plymouth - L4	UK	Atlantic Ocean	1,373	3.7
OSD2	0	Roscoff - SOMLIT	France	Atlantic Ocean	2,606	6.0
OSD3	0	Helgoland	Germany	Atlantic Ocean	1,129	2.8
OSD8	0	BATS	Bermuda	Atlantic Ocean	156	0.7
OSD14	2	Banyuls	France	Mediterranean Sea	740	2.1
OSD17	3	VLIZ	Belgium	Atlantic Ocean	179	0.6
OSD22	1	Marseille Solemio SOMLIT	France	Mediterranean Sea	120	0.2
OSD25	0	Saidia Rocher	Morocco	Mediterranean Sea	120	0.5
OSD26	0	Tangier	Morocco	Atlantic Ocean	1,205	3.6
OSD29	0	Florida	USA	Atlantic Ocean	917	3.3
OSD30	2	Tvarminne	Finland	Atlantic Ocean	1,097	2.4
OSD35	1	Cheasapeake Bay	USA	Atlantic Ocean	2,637	7.0
OSD36	0	Delaware	USA	Atlantic Ocean	1,282	4.0
OSD37	1	Port Everglades	USA	Atlantic Ocean	1,752	4.1
OSD38	0	Long Key	USA	Atlantic Ocean	256	0.8
OSD39	0	Charleston Harbor	USA	Atlantic Ocean	5,554	40.5
OSD43	0	SIO Pier	USA	Pacific Ocean	839	3.2
OSD47	0	Venice Lagoon	Italy	Mediterranean Sea	3,984	9.5
OSD50	0	Pasaia	Spain	Atlantic Ocean	177	0.7
OSD51	2	Bocas del Toro	USA	Atlantic Ocean	2,812	11.9
OSD54	1	Maine Booth Bay	USA	Atlantic Ocean	1,559	3.9
OSD55	1	Maine Damariscotta River	USA	Atlantic Ocean	1,307	3.7
OSD56	0	Hawaii Kakaako	Hawaii	Pacific Ocean	5,190	15.1
OSD57	0	Hawaii Oahu	Hawaii	Pacific Ocean	6,384	17.0
OSD58	1	PICO	USA	Atlantic Ocean	2,592	4.6
OSD60	0	South Carolina 2 - North Inlet	USA	Atlantic Ocean	1,852	4.3
OSD61	0	Vineyard Sound	USA	Atlantic Ocean	6,190	20.2
OSD62	0	Manai Straits	UK	Atlantic Ocean	465	1.2
OSD64	1	Odessa	Ukraine	Black Sea	1,507	2.7
OSD65	0	Leigh Marine Laboratory	New Zealand	Pacific Ocean	5,573	17.2
OSD69	0	Marghera	Italy	Mediterranean Sea	1,278	2.8
OSD70	0	Lido	Italy	Mediterranean Sea	2,728	6.1
OSD71	0	Otago	New Zealand	Pacific Ocean	7,662	13.7
OSD73	1	Lima Estuary	Portugal	Atlantic Ocean	571	2.2
OSD74	1	Douro Estuary	Portugal	Atlantic Ocean	102	0.2
OSD80	2	Young Sound	Greenland	Arctic Ocean	4,987	9.7
OSD81	0	Ria Formosa Lagoon	Portugal	Atlantic Ocean	837	4.6
OSD92	2	Casablanca	Morocco	Atlantic Ocean	11,606	27.5

OSD94	0	Saidia Marina	Morocco	Mediterranean Sea	155	0.7
OSD95	0	Singapore Indigo_V	Singapore	Pacific Ocean	1,960	3.1
OSD98	0	Sao Jorge Azores	Portugal	Atlantic Ocean	1,066	2.8
OSD101	0	Quinta do Lorde	Portugal	Atlantic Ocean	5,454	26.8
OSD102	0	Marina do Funchal	Portugal	Atlantic Ocean	9,598	16.9
OSD103	0	Porto da Cruz	Portugal	Atlantic Ocean	8,605	26.1
OSD105	4	Cambridge Bay, Nunavut	USA	Arctic Ocean	442	2.7
OSD106	0	REYKIS	Iceland	Atlantic Ocean	235	1.0
OSD106	15	REYKIS	Iceland	Atlantic Ocean	431	1.1
OSD111	0	Ria de Aveiro_1	Portugal	Atlantic Ocean	17,481	31.3
OSD113	39	CascaisWatch	Portugal	North Atlantic Ocean	570	2.6
OSD115	0	Santa Cruz	Portugal	Atlantic Ocean	261	2.0
OSD117	0	Tavira Beach	Portugal	Atlantic Ocean	2,852	7.2
OSD118	0	Lough Hyne	Ireland	Atlantic Ocean	6,354	14.6
OSD123	4	Shikmona	Israel	Mediterranean Sea	105	0.5
OSD124	5	Osaka Bay	Japan	Pacific Ocean	5,851	14.5
OSD125	0	Cullercoats Beach	UK	Atlantic Ocean	1,854	6.2
OSD126	1	Eyafjordur_1	Iceland	Arctic Ocean	265	0.7
OSD129	10	Eyafjordur_4	Iceland	Arctic Ocean	126	0.5
OSD130	1	Eyafjordur_5	Iceland	Arctic Ocean	1,432	3.1
OSD131	1	Zlatna ribka	Bulgaria	Black Sea	151	0.8
OSD132	0	Sdot YAM	Israel	Mediterranean Sea	795	4.0
OSD133	0	Robben Island	South Africa	Atlantic Ocean	3,613	10.3
OSD142	1	Gray's Reef Marine Sanctuary	USA	Atlantic Ocean	496	5.2
OSD143	1	Skidaway Institute	USA	Atlantic Ocean	2,167	4.8
OSD144	0	Maunalua Bay O'ahu	USA	Pacific Ocean	307	1.4
OSD145	0	Blankenberge	Belgium	Atlantic Ocean	956	1.9
OSD146	5	Fram Strait	Germany	Arctic Ocean	2,612	12.6
OSD147	0	Rajarata	Sri Lanka	Indian Ocean	3,707	7.6
OSD148	0	Wadden Sea	Germany	Atlantic Ocean	592	2.8
OSD149	0	Laguna Rocha Norte	Uruguay	Atlantic Ocean	4,433	11.4
OSD150	0	Laguna Rocha Sur	Uruguay	Atlantic Ocean	6,625	15.4
OSD152	1	Compass Buoy Station	Canada	Atlantic Ocean	171	0.5
OSD153	0	Faro Island	Portugal	Atlantic Ocean	821	2.9
OSD154	1	Arcachon-SOMLIT	France	Atlantic Ocean	3,752	10.5
OSD158	0	Sao Miguel Azores II	Portugal	Atlantic Ocean	316	1.8
OSD159	2	Brest-SOMLIT	France	Atlantic Ocean	302	1.5
OSD162	1	Stonehaven	UK	Atlantic Ocean	788	2.8
OSD163	2	Scapa	UK	Atlantic Ocean	2,625	12.9
OSD164	2	Scalloway	UK	Atlantic Ocean	227	3.1
OSD165	2	Loch Ewe	UK	Atlantic Ocean	208	1.4
OSD166	0	Armintza	Spain	Atlantic Ocean	5,246	21.0

OSD170	3	Belgium - 130	Belgium	Atlantic Ocean	3,098	4.7
OSD171	3	Belgium - 230	Belgium	Atlantic Ocean	519	1.4
OSD173	3	Belgium - 710	Belgium	Atlantic Ocean	796	1.4
OSD174	3	Belgium - 780	Belgium	Atlantic Ocean	131	0.2
OSD175	3	ZG02	Belgium	Atlantic Ocean	255	0.7
OSD176	3	Belgium - 215	Belgium	Atlantic Ocean	179	0.3
OSD177	3	Belgium - 120	Belgium	Atlantic Ocean	192	0.3
OSD178	3	Belgium - 435	Belgium	Atlantic Ocean	432	0.6
OSD182	3	W08	Belgium	Atlantic Ocean	1,009	2.9
OSD183	3	W09	Belgium	Atlantic Ocean	8,961	17.0
OSD184	3	W10	Belgium	Atlantic Ocean	1,632	3.4
OSD185	3	Belgium - 421	Belgium	Atlantic Ocean	2,099	3.2

Table S2. Matrix of the pairwise percent identity between *Ostreococcus* clades. The calculation was done based on the alignment available as Supplementary data S6.

Ostreococcus	"O. lucimarinus"	Clade B	O. tauri	O.mediterraneus	Clade E
"O. lucimarinus"	99.70	98.80	99.40	98.00	99.20
Clade B	98.80	99.70	99.10	97.70	99.40
O. tauri	99.40	99.10	99.70	98.30	99.50
O.mediterraneus	98.00	97.70	98.30	99.80	98.00
Clade E	99.20	99.40	99.50	98.00	99.80

Table S3. Matrix of the pairwise percent identity between *Micromonas* clades. The calculation was done based on the alignment available as Supplementary data S7.

Micromonas	M. pusilla	M. bravo B1	M. bravo B2	Clade B3	Clade B4	Clade B5	M. polaris	M. commoda A1	M. commoda A2
M. pusilla	99.20	96.80	97.00	96.30	96.70	98.20	96.90	96.60	97.20
M. bravo B1	96.80	99.40	99.30	97.80	96.90	97.90	98.30	97.60	97.40
M. bravo B2	97.00	99.30	100.00	98.40	97.20	98.10	98.90	97.70	97.50
Clade B3	96.30	97.80	98.40	100.00	96.90	97.20	99.10	97.20	97.00
Clade B4	96.70	96.90	97.20	96.90	99.80	97.80	97.50	97.40	97.60
Clade B5	98.20	97.90	98.10	97.20	98.70	99.80	97.60	97.90	98.30
M. polaris	96.90	98.30	98.90	99.10	97.50	97.60	99.90	97.30	97.00
M. commoda A1	96.60	97.60	97.70	97.20	97.40	97.90	97.30	100.00	99.40
M. commoda A2	97.20	97.40	97.50	97.00	97.60	98.30	97.00	99.40	99.90

Table S4. Matrix of the pairwise percent identity between *Mantoniella* clades. The calculation was done based on the alignment available as Supplementary data S8.

Mantoniella	M. antarctica	M. squamata	M. clade A	M. clade B
M. antarctica	99.70	99.00	96.70	99.30
M. squamata	99.00	100.00	97.50	99.80
M. clade A	96.70	97.50	97.60	97.30
M. clade B	99.30	99.80	97.30	99.90

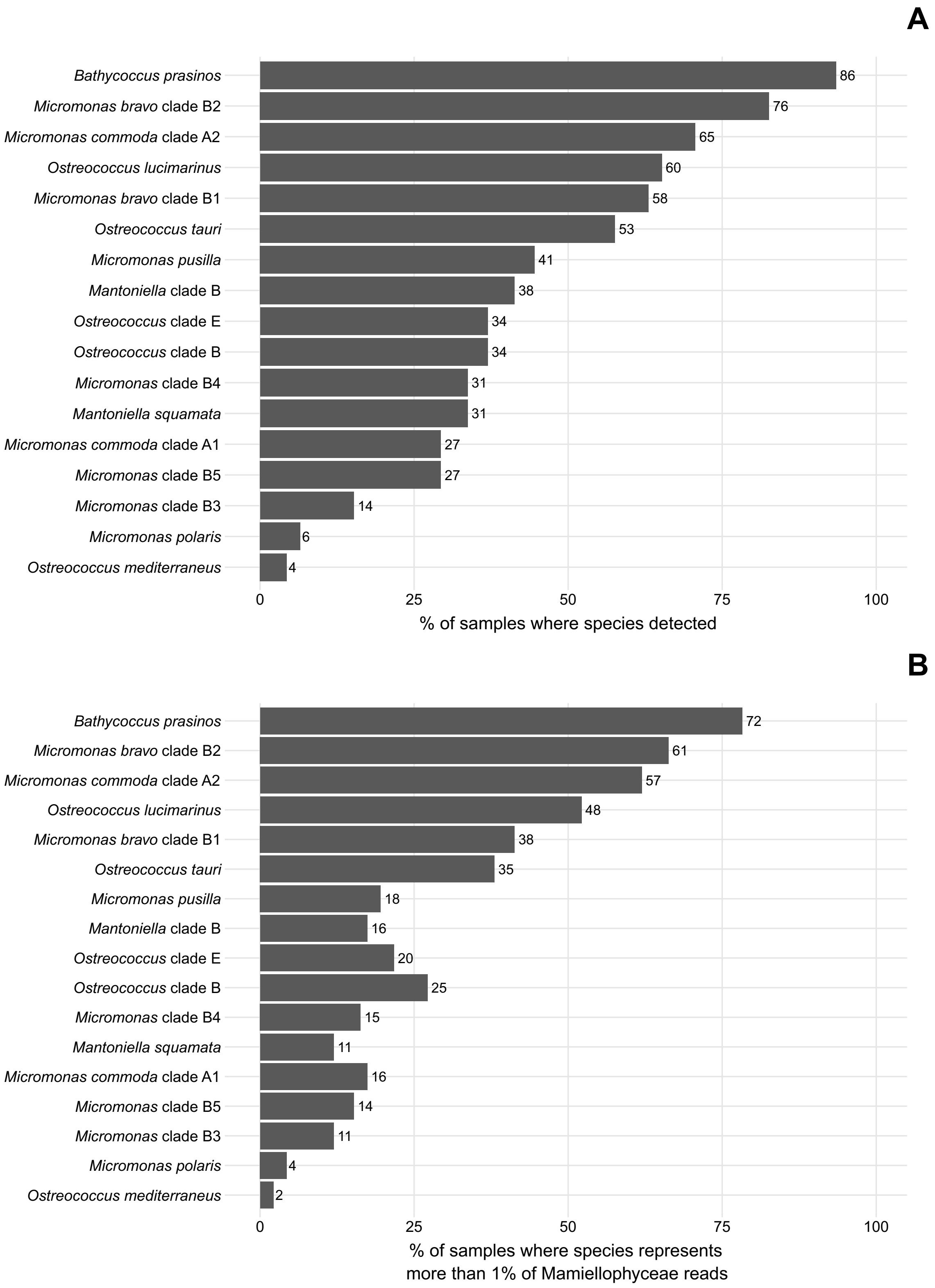

Figure S1. A. Percentage of OSD samples where a given ASV was detected (at least one read). Numbers at right of bars correspond to number of samples. B. Idem but for samples where the ASV contributed more than 1% of the Mamiellophyceae reads. Stations with less than 100 Mamiellophyceae reads were not considered. The three major *O. tauri* ASVs have been pooled together.

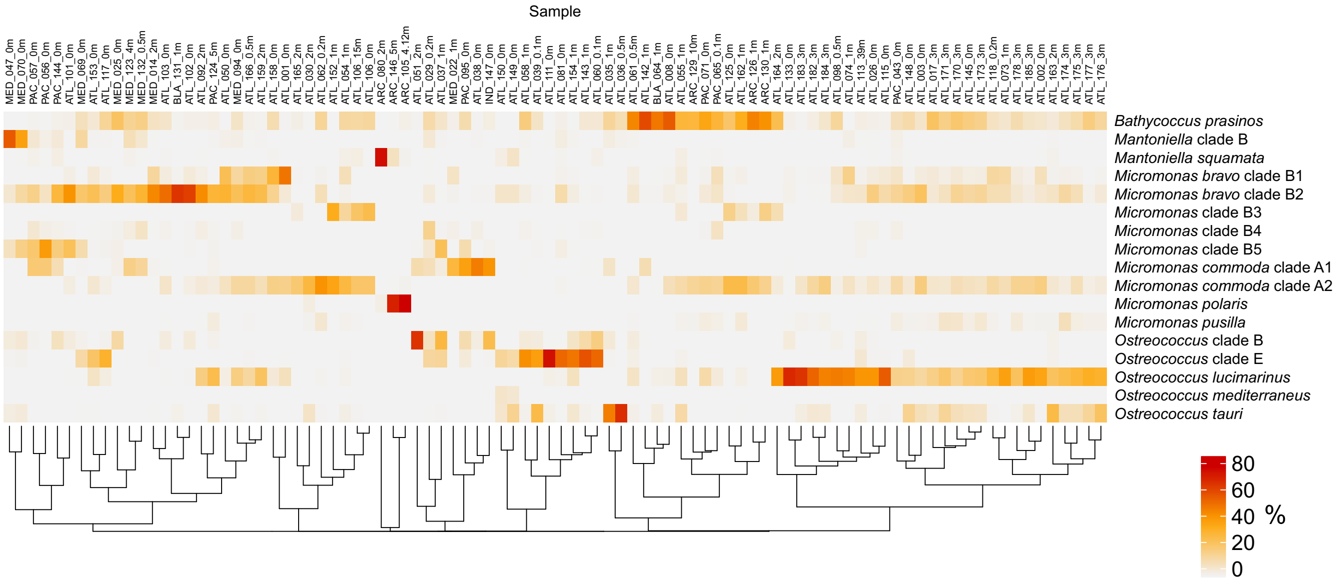

Figure S2. Heatmap of the Mamiellophyceae communities in OSD samples. The number of read corresponding to the major ASVs were normalized by the number of Mamiellophyceae reads per station. Stations with less than 100 Mamiellophyceae reads were not considered.

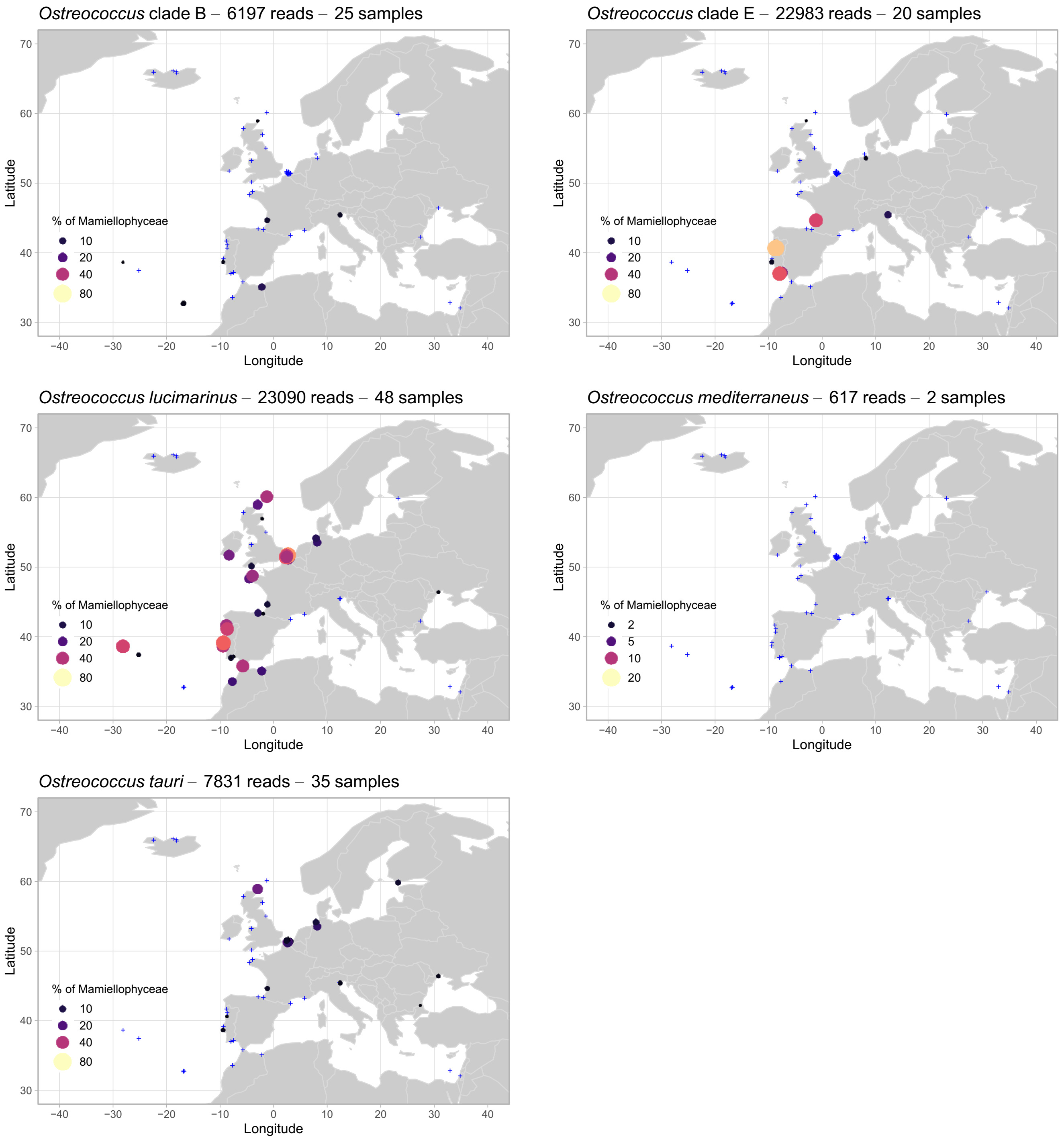

Figure S3. Distribution of major *Ostreococcus* ASVs for OSD2014 (LGC) in European waters. The three major O. tauri ASVs have been pooled together. Circle surface corresponds to the percentage of ASV reads relative to the total number of Mamiellophyceae reads. Samples for which the ASV contribution was lower than 1 % are represented by blue crosses.

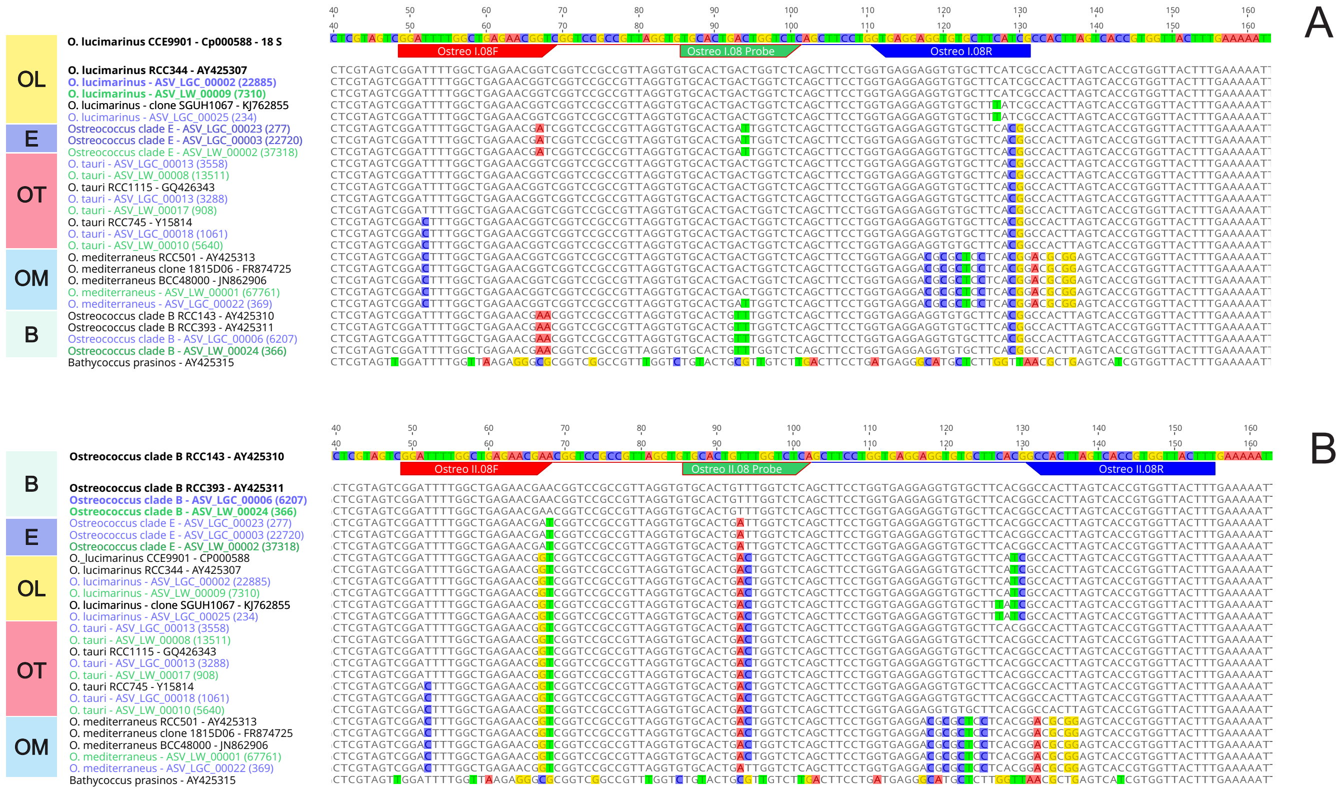

Figure S4. Alignments of *Ostreococcus* V4 regions and localization of the qPCR primers and probes (Demir-Hilton et al. 2011) used to quantify the OI (A) and OII (B) clades. Sequences with no mismatches to each probe set are in bold. Blue and green sequences correspond to LGC and LW datasets, respectively.

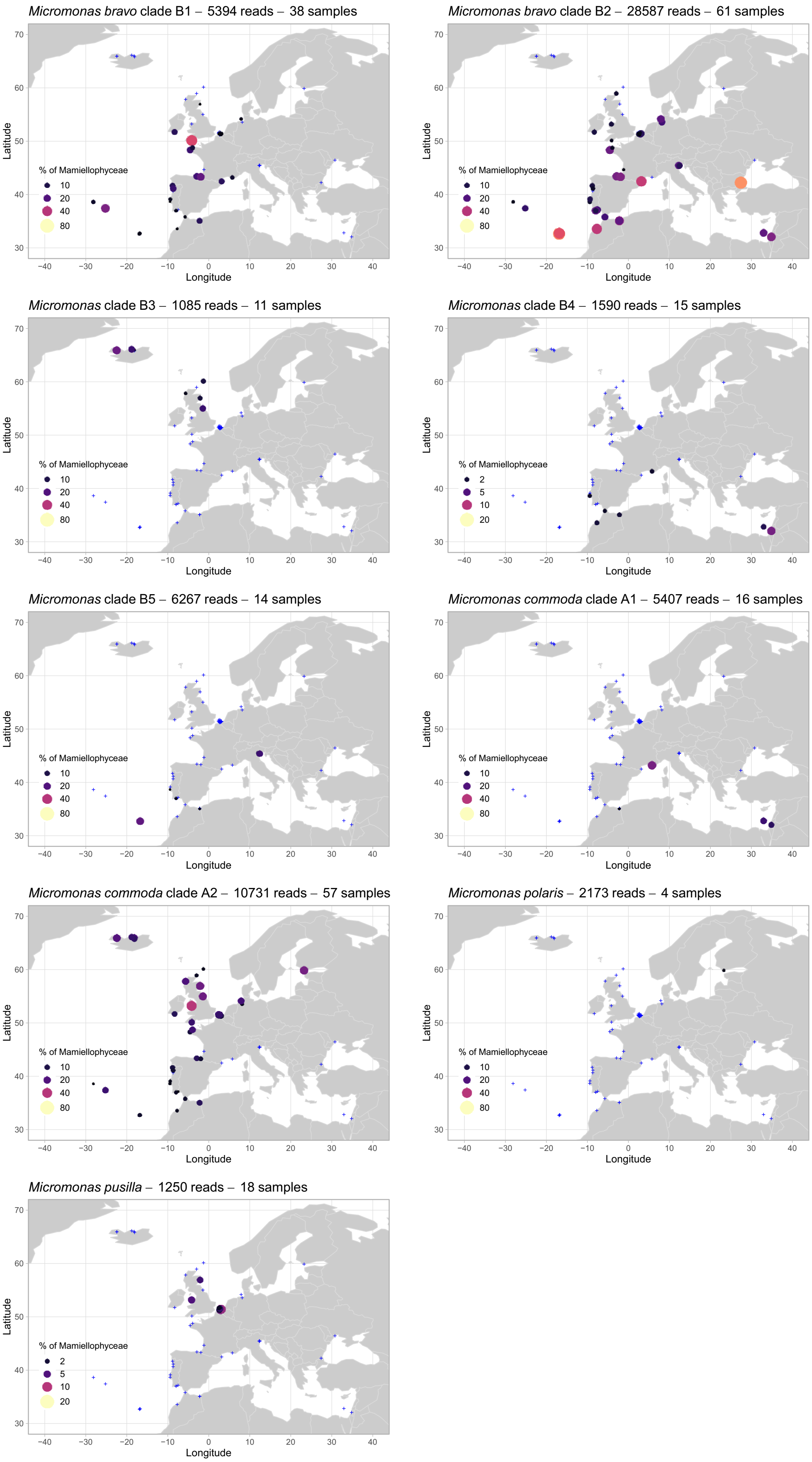

Figure S5. Same as Fig. S3 for *Micromomas*.

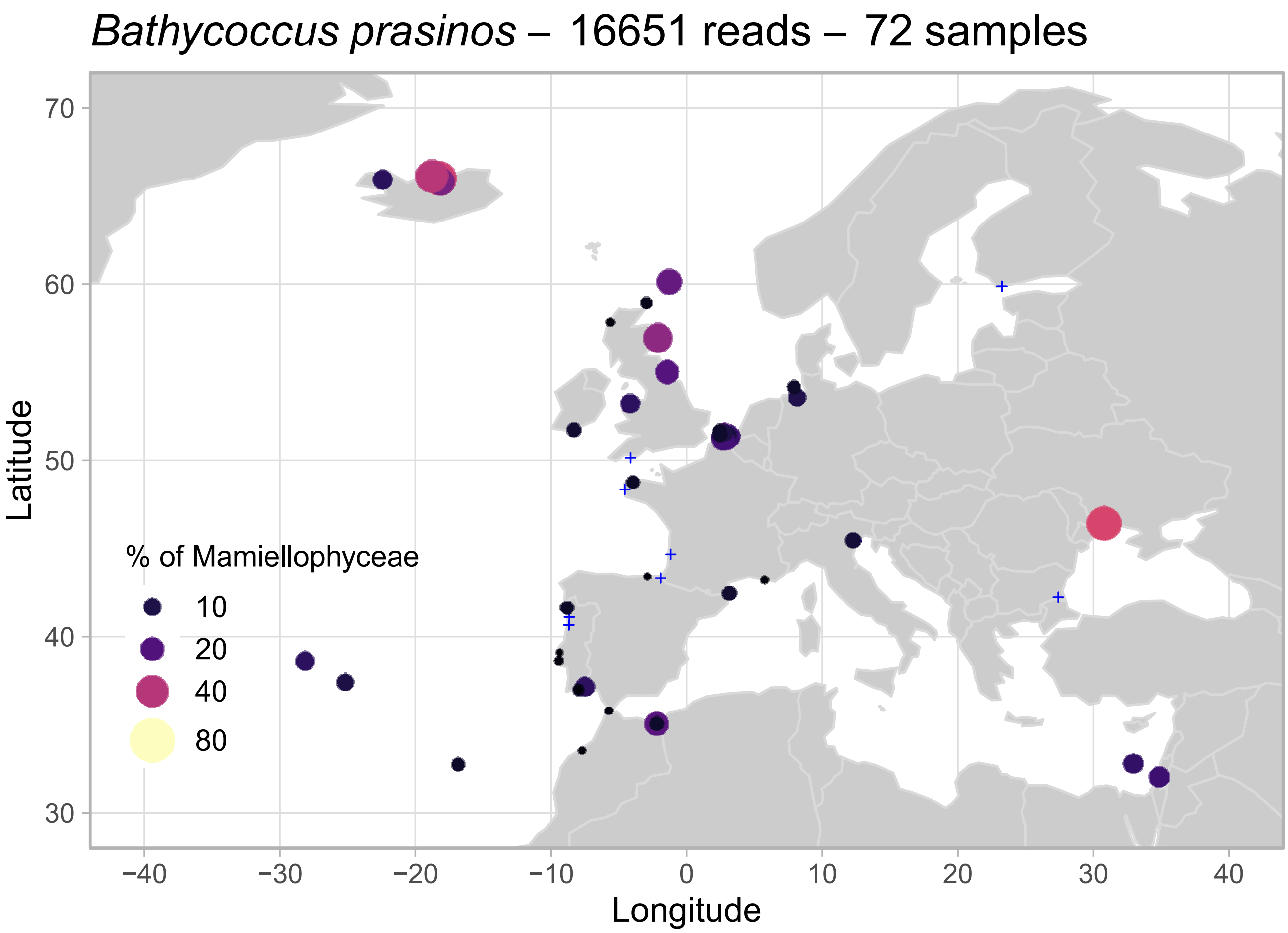

Figure S6. Same as Fig. S3 for *Bathycoccus*.

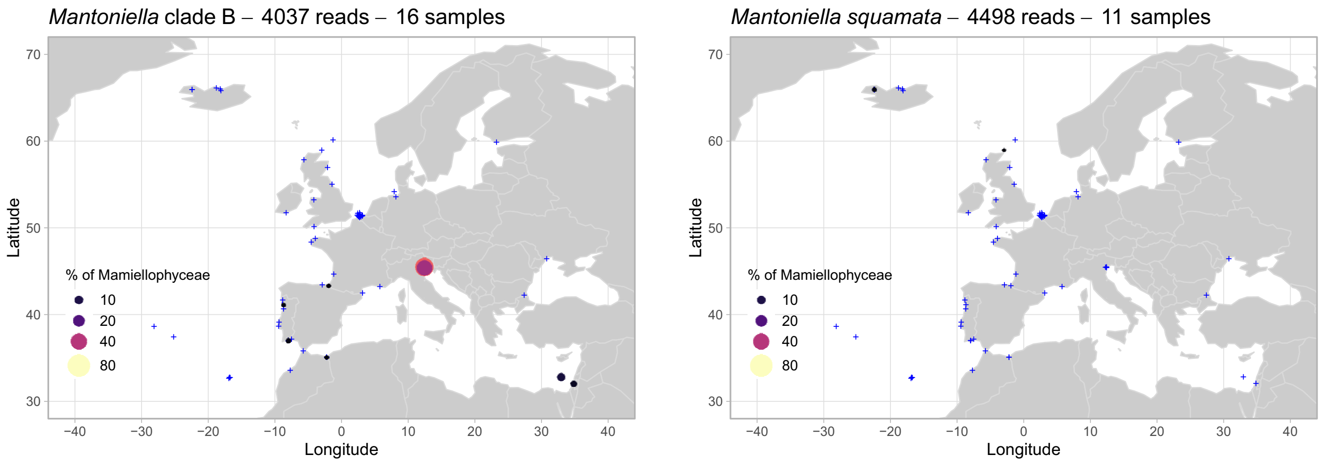

Figure S7. Same as Fig. S3 for *Mantoniella*.
